# Correlation Investigation between Pyrrole-DNA and Pyrrole-Protein Adducts in Male ICR Mice Exposed to Retrorsine, a Hepatotoxic Pyrrolizidine Alkaloid

**DOI:** 10.3390/toxins14060377

**Published:** 2022-05-28

**Authors:** Lin Zhu, Junyi Xue, Yisheng He, Qingsu Xia, Peter P. Fu, Ge Lin

**Affiliations:** 1 School of Biomedical Sciences, Faculty of Medicine, The Chinese University of Hong Kong, Hong Kong, China; linzhu912@gmail.com (L.Z.); junyi.xue@gmail.com (J.X.); yshe@link.cuhk.edu.hk (Y.H.); 2 National Center for Toxicological Research, U.S. Food and Drug Administration, Jefferson, AR 72079, USA; qingsu.xia@fda.hhs.gov

**Keywords:** pyrrolizidine alkaloids, retrorsine, pyrrole-protein adducts, pyrrole-DNA adducts, biomarker

## Abstract

Pyrrolizidine alkaloids (PAs) have been found in over 6000 plants worldwide and represent the most common hepatotoxic phytotoxins. Catalyzed by hepatic cytochrome P450 enzymes, PAs are metabolized into reactive pyrrolic metabolites, which can alkylate cellular proteins and DNA to form pyrrole-protein adducts and pyrrole-DNA adducts, leading to cytotoxicity, genotoxicity, and tumorigenicity. To date, the correlation between these PA-derived pyrrole-protein and pyrrole-DNA adducts has not been well investigated. Retrorsine is a representative hepatotoxic and carcinogenic PA. In the present study, the correlations among the PA-derived liver DNA adducts, liver protein adducts, and serum protein adducts in retrorsine-treated mice under different dosage regimens were studied. The results showed positive correlations among these adducts, in which serum pyrrole-protein adducts were more accessible and present in higher abundance, and thus could be used as a suitable surrogate biomarker for pyrrole-DNA adducts to indicate the genetic or carcinogenic risk posed by retrorsine.

## 1. Introduction

Pyrrolizidine alkaloids (PAs) are phytotoxins produced by more than 6000 plant species of 13 distant families [1,2]. Approximately 500–600 potentially poisonous PAs and PA *N*-oxides have been identified in about 3–5% of the world’s floriferous plants [1,3]. Humans are readily exposed to toxic PAs via the consumption of PA-producing plants used in herbal medicines, herbal teas, dietary supplements, and/or PA-contaminated staple foods [4,5]. The transfer of PAs through the food chain from livestock to dietary foodstuffs, such as milk, eggs, meats, and honey, significantly increases the chances of PA exposure in humans [6,7,8,9,10]. 

**Figure 1:**
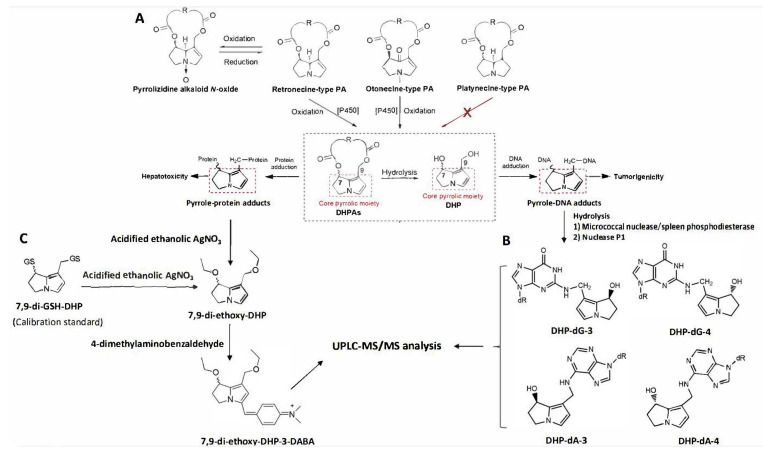
Metabolic activation of toxic PAs and PA *N*-oxides to induce hepatotoxicity and tumorigenicity (**A**), scheme of enzymatic hydrolysis and analysis of pyrrole-DNA adducts (**B**), and scheme of precolumn derivatization and analysis of pyrrole-protein adducts (**C**).

PAs are esters formed by a necine base and one or two necic acids. Based on the necine base structure, PAs are generally classified into three types: retronecine (including its 7-α enantiomer, heliotridine), otonecine, and platynecine [1,11]. The retronecine and otonecine types of PAs possessing unsaturated necine bases are hepatotoxic, while platynecine-PAs with saturated necine bases are non-toxic. Retronecine-type PAs are present in many plants, such as various species in genus of *Asmachila*, *Heliotropium*, *Comfrey*, *Gynura*, *Senecio*, etc. [1,5,12], and also found to exist in plants in both tertiary base form and *N*-oxide form [13,14,15]. Catalyzed by hepatic cytochrome P450 monooxygenases, the metabolism of retronecine-type and otonecine-type PAs, but not platynecine-type PAs, forms the corresponding reactive pyrrolic metabolites, dehydropyrrolizidine alkaloids (DHPAs) [1,2,11,16,17]. DHPAs are chemically and biologically reactive and can rapidly hydrolyze to the less reactive (±)-6,7-dihydro-7-hydroxy-1-hydroxymethyl-5*H*-pyrrolizine (DHP). Both DHPAs and DHP can react with nearby macromolecules such as DNA and proteins to form a series of pyrrolic adducts [1,4] (Figure 1A). Not only are these biochemical events relevant to the mechanisms of PA-induced toxicity, but the detection of adducts provides a means of monitoring exposure to toxic PAs.

The PA-derived DNA adducts were firstly characterized in a PA-exposed rat model by Fu et al. in 2010 with full structures elucidated as a pair of epimers of 7-hydroxy-9-(deoxyadenosin-*N*6-yl) dehydrosupinidine (named DHP-dA-3 and DHP-dA-4) and a pair of epimers of 7-hydroxy-9-(deoxyguanosin-*N*2-yl)dehydrosupinidine (named DHP-dG-3 and DHP-dG-4) [18] (Figure 1B). These identified pyrrole-DNA adducts, although they have not been detected in biological liver specimens, are found as a mechanism-based common biological biomarker of PA-induced tumorigenicity [19]. On the other hand, DHPAs, as reactive electrophiles, can also bind to nucleophilic sites of proteins to form pyrrole-protein adducts (Figure 1C). Pyrrole-protein adducts were first detected in the serum of PA-induced liver injury patients who consumed a PA-containing herb by our group in 2011 [12]. Since then, pyrrole-protein adducts were unequivocally detected in the specimens of both liver and serum from PA-treated rats, demonstrating that pyrrole-protein adducts can be used as a biomarker for PA-induced hepatotoxicity [20,21].

Apparently, both PA-derived DNA and protein adducts can serve as useful biomarkers for detecting human exposure to toxic PAs. So far, PA-derived DNA and protein adducts are studied separately [20,22,23,24], while whether there is a correlation between the formation of these adducts is unknown. Considering the clinical practice, exploration of correlations between PA-derived DNA and protein adducts can potentially develop the use of blood pyrrole-protein adducts as a surrogate biomarker of pyrrole-DNA adducts to correlate with PA-induced tumorigenicity. Retrorsine is a commonly studied hepatotoxic and carcinogenic PA. To pursue this goal, in the present study, we conducted the correlation investigation of retrorsine-derived adducts, including liver pyrrole-DNA adducts, liver pyrrole-protein adducts, and serum pyrrole-protein adducts, formed in retrorsine-treated mice. 

## 2. Materials and Methods

Procedures involving the care and handling of mice were reviewed and approved by the Animal Experimental Ethics Committee, the Chinese University of Hong Kong (CUHK), under the regulations of the Hong Kong SAR government. Male ICR mice (20–25 g) supplied by the Laboratory Animal Services Center at CUHK were randomly divided into different groups (*n* = 4 mice per group) for the treatment of retrorsine, a representative toxic PA to induce liver injury in a mouse model [12,24,25,26] and vehicle control. For the dose-response experiment, mice in different groups were treated orally with a single dose of retrorsine at 10, 20, 40, or 60 mg/kg b.w. and sacrificed at 24 h after the treatment. For the kinetic investigation experiment, mice in different groups were orally administrated with a single dose of 40 mg/kg b.w. of retrorsine and sacrificed at 2, 4, 6, 8, and 12 h, 1, 2, and 3 days, or 1, 2, 4, and 8 weeks after the treatment. For both studies, mice treated in parallel with water served as controls. After the sacrifice by overexposure to carbon dioxide, blood and liver samples were collected. Blood samples were immediately centrifuged at 2000× *g* for 10 min at 4 °C, and serum samples were harvested and stored at −80 °C for further analysis of serum pyrrole-protein adducts. Liver tissues were stored at −80 °C until the analysis of liver pyrrole-protein and pyrrole-DNA adducts. 

The liver and serum pyrrole-protein adducts were quantified using our previously established ultraperformance liquid chromatography-tandem mass spectrometry (UPLC-MS/MS) method with a pre-column derivatization [27]. Briefly, individual serum (100 µL) or liver samples (50 mg) were mixed with 500 µL acetone and centrifuged at 900 g for 5 min. The precipitated pellets were washed with absolute ethanol (500 µL) and reacted with 100 µL of 2% silver nitrate ethanol solution containing 5% trifluoroacetic acid for 30 min under shaking. The resultant supernatant was reacted with 4-dimethylaminobenzaldehyde (*v*/*v*, 4:1) in ethanol containing 1% perchloric acid at 55 °C for 10 min to produce the pyrrole-derived analyte which was further analyzed by UPLC–MS/MS under multiple reaction monitoring modes with the transition of *m*/*z* 341.2 > 252.2. A pyrrole–glutathione conjugate (7,9-diglutathione-(±)-6,7-dihydro-7-hydroxy-1-hydroxymethyl-*5H*-pyrrolizine) was used as a standard (Figure 1C) and underwent the identical derivatization and UPLC–MS/MS analysis for the construction of a calibration curve.

Quantitation of liver pyrrole-DNA adducts was conducted according to our previously reported method [18,19]. Liver DNA was extracted using a Blood & Cell Culture DNA Isolation kit (QIAGEN Inc., Valencia, CA, USA) and enzymatically hydrolyzed to nucleosides with micrococcal nuclease, spleen phosphodiesterase, and nuclease P1 as previously reported. The internal standards (DHP-[^15^N_5_]dG-3, DHP-[^15^N_5_]dG-4, DHP-[^15^N_5_,^13^C_10_]dA-3 and DHP-[^15^N_5_,^13^C_10_]dA-4) (Figure 1B) were added into the hydrolyzed solution, and a 20-µL aliquot was injected into the UPLC-MS/MS for the analysis of pyrrole-DNA adducts. The DHP-dG adducts were monitored at the [M + H] + m/z 403 to [M + H − 134]^+^ *m*/*z* 269 transition and the DHP-[^15^N_5_]dG internal standards at the [M + H] + *m*/*z* 408 to [M + H − 134]^+^ *m*/*z* 274 transition. The DHP-dA adducts were monitored at the [M + H] + *m*/*z* 387 to [M + H − 134]^+^ *m*/*z* 253 transition, and the DHP-[^15^N_5_,^13^C_10_]dA internal standards at the [M + H]^+^ *m*/*z* 402 to [M + H − 139]^+^ *m*/*z* 263 transition. The calibration curves were generated for synthetically prepared DHP-dG-3, DHP-dG-4, DHP-dA-3, and DHP-dA-4 versus their respective DHP-[^15^N_5_]dG and DHP-[^15^N_5_,^13^C_10_]dA internal standards. Samples were quantified by comparing the areas of the unlabeled chromatogram peaks to those of the corresponding labeled internal standard chromatogram peaks. The levels of pyrrole-DNA adducts were calculated as a sum of DHP-dG and DHP-dA adducts. All data are expressed as mean ± SEM. Statistical analyses were performed by Prism 6.0 (GraphPad Software Inc., San Diego, CA, USA).

## 3. Results

**Figure 2:**
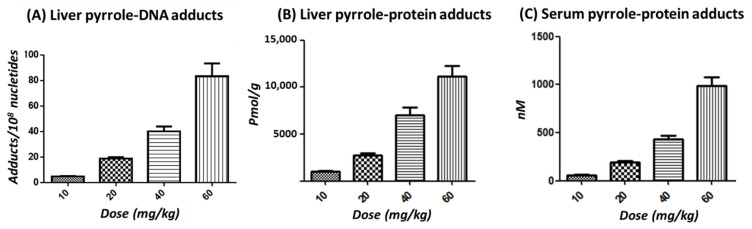
Levels of liver pyrrole-DNA adducts (**A**), liver pyrrole-protein adducts (**B**), and serum pyrrole-protein adducts (**C**) formed in male ICR mice at 24 h after a single oral dose of 10, 20, 40, or 60 mg/kg of retrorsine.

**Figure 3:**
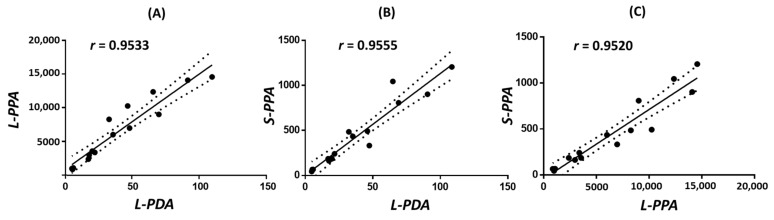
Correlation results (**A**–**C**) with Pearson’s correlation coefficient (*r*) between levels of liver pyrrole-DNA adducts (L-PDA), liver pyrrole-protein adducts (L-PPA), and serum pyrrole-protein adducts (S-PPA) formed in male ICR mice at 24 h after a single oral dose of 10, 20, 40, or 60 mg/kg of retrorsine.

To investigate the dose-response relationship among different retrorsine-treated groups, mice were orally gavaged with a single dose of vehicle or 10, 20, 40, or 60 mg/kg of retrorsine. Liver injury, manifested by hepatic sinusoidal hemorrhage, was observed in mice dosed with 40 mg/kg and 60 mg/kg of retrorsine [24]. After a 24-h treatment, liver pyrrole-DNA adducts, liver pyrrole-protein adducts, and serum pyrrole-protein adducts were all detected in retrorsine-treated groups with increased formation in a dose-dependent manner but were absent in the vehicle control group (Figure 2A–C, Table S1 and Figures S1 and S2). Pearson’s analysis was used to assess correlations among three types of adducts as moderate, strong, or very strong correlations if the Pearson correlation coefficient (r) was between 0.40 and 0.69, 0.70 and 0.89, or 0.90 and 0.99, respectively. Significantly positive correlations have been found in each of the comparisons, indicating that they were closely related over the investigated dose range (Figure 3).

**Figure 4:**
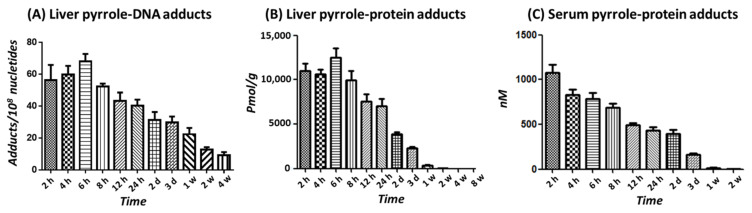
Levels of liver pyrrole-DNA adducts (**A**), liver pyrrole-protein adducts (**B**), and serum pyrrole-protein adducts (**C**) formed in male ICR mice at different time points after a single oral dose of 40 mg/kg of retrorsine.

**Figure 5:**
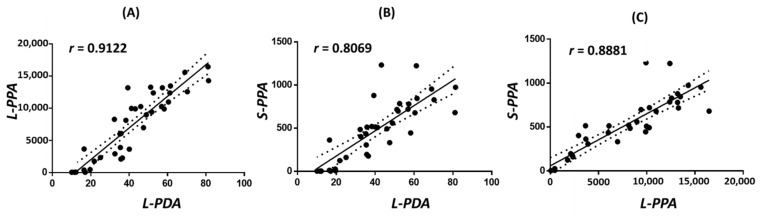
Correlation results (**A**–**C**) with Pearson’s correlation coefficient (*r*) between levels of liver pyrrole-DNA adducts (L-PDA), liver pyrrole-protein adducts (L-PPA), and serum pyrrole-protein adducts (S-PPA) formed in male ICR mice at different time points after a single oral dose of 40 mg/kg of retrorsine.

Further, kinetics for the series of retrorsine-derived adducts investigated in the mice model were orally administered with a single dose of 40 mg/kg retrorsine and sacrificed at 2, 4, 6, 8, and 12 h, or 1, 2, and 3 days, or 1, 2, 4, and 8 weeks after dosing. The results demonstrated that both pyrrole-DNA and pyrrole-protein adducts were quickly formed in the liver, and the maximum concentrations (*T*_max_) were reached at about 6 h after the treatment (Figure 4A,B and Table S2). Afterwards, the decline followed a biphasic elimination pattern, with a fast initial phase followed by an extended slower phase, suggesting sufficient persisting time in the liver. The persistent time for liver pyrrole-DNA adducts and pyrrole-protein adducts has been observed to be 4 and 8 weeks, respectively. While for the pyrrole-protein adducts determined in the serum, without the peak levels observed, the adducts started decreasing initially and remained detectable until 2 weeks post-dosing (Figure 4C and Table S2). On the other hand, the removal of the serum pyrrole-protein adducts exhibited a one-phase profile, which was synchronous with the fast elimination phase of liver pyrrole-protein adducts. We determined the Pearson’s correlation coefficient for each of the comparisons. Similar to the dose-response experiment, there were strong, statistically significant Pearson’s correlations among them (Figure 5).

Taken together, in the present study, the correlation results showed that the binding of DHPA to liver and serum proteins was proportional to its binding to liver DNA. As protein binding is more abundant and can be found in more easily accessible fluids such as blood, it might be possible to estimate DNA binding through the measurement of protein binding.

## 4. Discussion

The continuous accumulation of evidence for the harm of PAs to human health necessitates the development and application of robust biomarkers for PA exposure. Characterization of the different types of PA-derived protein and DNA adducts has not only determined the mechanisms of PA-induced toxicities but also potentially provides a means for exposure monitoring and risk assessment. The formation of PA-derived DNA adducts is considered a critical step in the initiation of PA-induced tumorigenicity, which, therefore, could serve as risk-associated biomarkers for early prediction of cancer risk [28,29]. However, the analysis of DNA adducts has limitations in terms of either accessibility or availability of DNA samples from human biological specimens. As a more abundant cellular constituent, protein adducts can thus serve as a useful surrogate biomarker of DNA adducts. Unlike DNA adducts, which can be removed by repair mechanisms, protein adducts are not subject to repair, are apparently stable for the lifetime of different proteins, are more easily found in accessible fluids such as blood, are present in adequate concentrations, and therefore could be considered in the estimation of genetic or carcinogenic risk whenever they can be correlated with DNA binding. 

The correlation analysis conducted in the present study showed strong correlations between each pair of the investigated adducts, indicating their molecular events may run in parallel or in a chain of consecutive events. It is known that all toxic PAs undergo metabolic activation to generate DHPAs, which possess the same core structure (pyrrolic moiety) regardless of the original structures of the PAs. DHPAs are chemically and biologically reactive, capable of alkylating cellular proteins and DNAs to form protein adducts and DNA adducts [1,2]. In the present study, extraction of DNA and protein from separate aliquots of liver tissue and comparison of DNA and protein adduct levels did show a very strong correlation, further supporting that they were formed in parallel and not in a competitive process. 

For the serum protein adducts, little is known about the mechanisms of pyrrole-protein adducts released into circulation. Some in vitro experiments suggest that this may involve multiple mechanisms, including secretion of adducted proteins and diffusion of DHPAs directly into the plasma. For instance, pyrrole-protein adducts were detected in the medium of human hepatic sinusoidal endothelial cells (HSEC) and human HepaRG hepatocytes cultured in the absence of extracellular protein, suggesting that pyrrole-protein adducts are actively secreted out of hepatocytes into the extracellular space [30,31,32,33]. On the other hand, DHPAs were found to exist in the blood efflux from the hepatic veins of PA-exposed rats, indicating that DHPAs could diffuse out of the hepatocyte and bind covalently to extra-hepatic proteins [34]. Not only detectable in the serum of PA-induced hepatotoxicity patients [35], pyrrole-protein adducts were also detected in the serum of PA treated mice, even with low doses of PAs which do not cause liver injury [16]. Therefore, the measurement of pyrrole-protein adducts in serum can potentially be used as a more practical biomarker for PA intoxication. 

In conclusion, by using retrorsine, a representative hepatotoxic and carcinogenic PA, the present study provided the general phenomena on the correlations among the PA-derived DNA and protein adducts, which fills a knowledge gap in this vitally important but largely unexplored research field for biomarker studies. With the significantly positive correlation between liver pyrrole-DNA and pyrrole-protein adducts, the pyrrole-protein adducts which appeared in the serum could be considered a useful indicator for PA-induced hepatotoxicity as well as liver cancer risk. Further research is required in order to make progress in understanding the molecular background of the correlations among these biomarkers and to apply this tool successfully for human genotoxic risk prediction.

## Supplementary Materials

The following supporting information can be downloaded at: https://www.mdpi.com/article/10.3390/toxins14060377/s1, Figure S1: Representative MRM chromatograms of UPLC-MS/MS analysis of liver pyrrole-DNA adducts in (a) control mice and (b) mice dosed with 20 mg/kg RTS for 24 h. Individual pyrrole-DNA adducts or internal standards were monitored as indicated in each channel. Figure S2: Representative MRM chromatograms of UPLC-MS/MS analysis of liver pyrrole-protein adducts in (a) control mice and (b) mice dosed with 20 mg/kg RTS for 24 h; and serum pyrrole-protein adducts in (c) control mice and (d) mice dosed with 20 mg/kg RTS for 24 h. Table S1: Levels of liver pyrrole-DNA adducts, liver pyrrole-protein adducts, and serum pyrrole-protein adducts formed in male ICR mice at 24 h after a single oral dose of 10, 20, 40, or 60 mg/kg of retrorsine. Table S2: Levels of liver pyrrole-DNA adducts, liver pyrrole-protein adducts, and serum pyrrole-protein adducts formed in male ICR mice at different time points after a single oral dose of 40 mg/kg of retrorsine.
