# Supplementary material for: Correlation Investigation between Pyrrole-DNA and Pyrrole-Protein Adducts in Male ICR Mice Exposed to Retrorsine, a Hepatotoxic Pyrrolizidine Alkaloid
Source: Toxins (Basel). 2022 May 28;14(6):377. doi: 10.3390/toxins14060377 (PMC9231038; doi:10.3390/toxins14060377)
Supplement: Supplementary file 1 [file toxins-14-00377-s001.zip › toxins-1739025-supplementary.pdf]

toxins

Supplementary materials

Correlation Investigation between Pyrrole-DNA and Pyrrole-Protein Adducts in Male ICR Mice Exposed to Retrorsine, a Hepatotoxic Pyrrolizidine Alkaloid

Lin Zhu [1], Junyi Xue [1], Yisheng He [1], Qingsu Xia [2], Peter P. Fu [2,*] and Ge Lin [1,*]

[1] School of Biomedical Sciences, Faculty of Medicine, The Chinese University of Hong Kong, Hong Kong, China; linzhu912@gmail.com (L.Z.); junyi.xue@gmail.com (J.X.); yshe@link.cuhk.edu.hk (Y.H.)
[2] National Center for Toxicological Research, U.S. Food and Drug Administration, Jefferson, AR 72079, USA; qingsu.xia@fda.hhs.gov
* Correspondence: peter.fu@fda.hhs.gov (P.P.F.); linge@cuhk.edu.hk (G.L.)

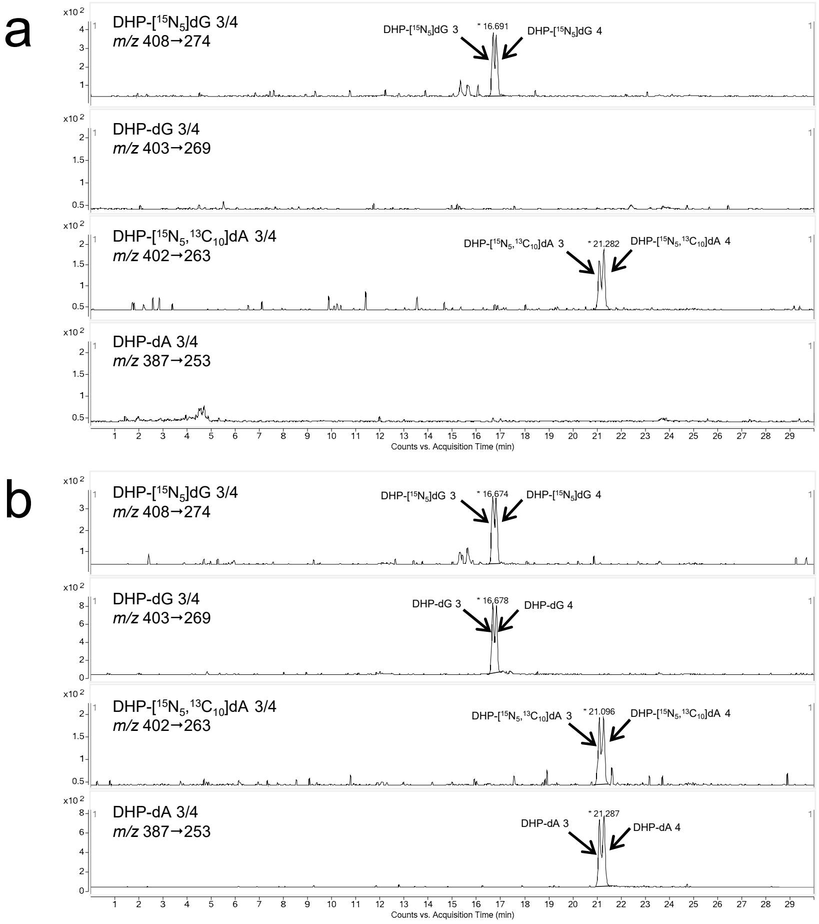

Figure S1 Representative MRM chromatograms of UPLC-MS/MS analysis of liver pyrrole-DNA adducts in (a) control mice and (b) mice dosed with 20 mg/kg RTS for 24 h. Individual pyrrole-DNA adducts or internal standards were monitored as indicated in each channel.

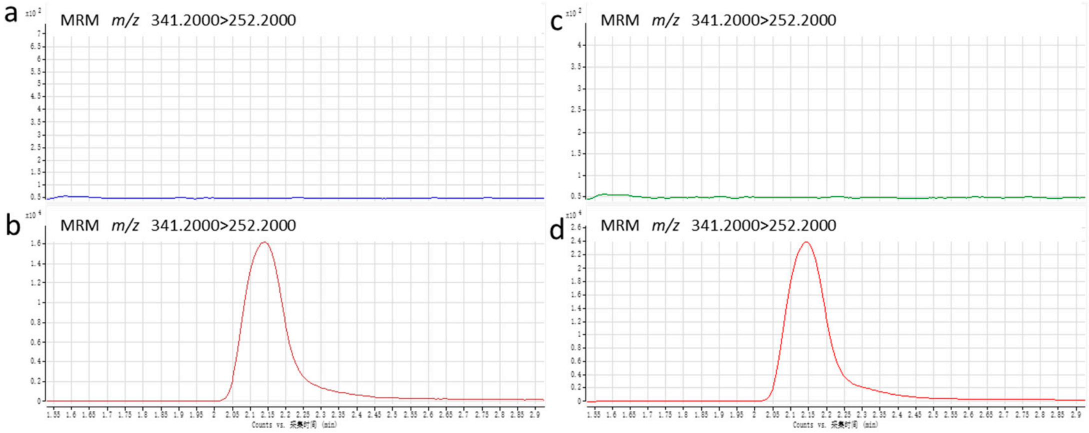

Figure S2 Representative MRM chromatograms of UPLC-MS/MS analysis of liver pyrrole-protein adducts in (a) control mice and (b) mice dosed with 20 mg/kg RTS for 24 h; and serum pyrrole-protein adducts in (c) control mice and (d) mice dosed with 20 mg/kg RTS for 24 h.

Table S1 Levels of liver pyrrole-DNA adducts, liver pyrrole-protein adducts, and serum pyrrole-protein adducts formed in male ICR mice at 24 h after a single oral dose of 10, 20, 40, or 60 mg/kg of retrorsine.

Dose (mg/kg)	Liver pyrrole-DNA adducts (adducts/10^8 nucletides)	Liver pyrrole-protein adducts (pmol/g)	Serum pyrrole-protein adducts
10	4.98±0.64	1055.61±104.44	58.76±12.50
20	18.99±2.18	2731.77±471.13	193.72±33.15
40	40.25±7.61	7011.28±1634.23	435.65±73.74
60	83.26±20.25	11161.83±2289.52	989.98±173.97

Table S2 Levels of liver pyrrole-DNA adducts, liver pyrrole-protein adducts, and serum pyrrole-protein adducts formed in male ICR mice at different time points after a single oral dose of 40 mg/kg of retrorsine.

Time	Liver pyrrole-DNA adducts (adducts/108 nucletides)	Liver pyrrole-protein adducts (pmol/g)	Serum pyrrole-protein adducts (nM)
2 h	56.25±19.26	10997.13±1619.06	1076.61±178.56
4 h	59.93±9.22	10629.76±1070.24	832.39±118.92
6 h	68.03±9.52	12512.72±2135.39	788.48±134.97
8 h	52.31±3.57	9955.98±2051.89	687.60±93.25
12 h	43.40±10.15	7557.52±1616.30	495.69±34.72
24 h	40.25±7.61	7011.28±1634.23	435.65± 73.74
2 d	31.15±10.23	3839.34±452.36	395.86±87.65
3 d	29.73±7.44	2283.30±278.36	164.39±30.17
1 w	22.37±7.82	377.86±99.18	14.68±9.41
2 w	12.74±3.03	44.32±6.19	3.26±0.43
4 w	9.35±3.65	6.07±1.18	<LOQ
8 w	<LOQ	2.66±0.15	<LOQ

LOQ: limit of quantitation.
